# Inflammation Aggravates Disease Severity in Marfan Syndrome Patients

**DOI:** 10.1371/journal.pone.0032963

**Published:** 2012-03-30

**Authors:** Teodora Radonic, Piet de Witte, Maarten Groenink, Vivian de Waard, Rene Lutter, Marco van Eijk, Marnix Jansen, Janneke Timmermans, Marlies Kempers, Arthur J. Scholte, Yvonne Hilhorst-Hofstee, Maarten P. van den Berg, J. Peter van Tintelen, Gerard Pals, Marieke J. H. Baars, Barbara J. M. Mulder, Aeilko H. Zwinderman

**Affiliations:** 1 Department of Clinical Epidemiology, Biostatistics and Bioinformatics, Academic Medical Centre, Amsterdam, The Netherlands; 2 Department of Cardiology, Academic Medical Centre, Amsterdam, The Netherlands; 3 Interuniversity Cardiology Institute of the The Netherlands, Utrecht, The Netherlands; 4 Department of Radiology, Academic Medical Centre, Amsterdam, The Netherlands; 5 Department of Medical Biochemistry, Academic Medical Centre, Amsterdam, The Netherlands; 6 Department of Pulmonology and Experimental Immunology, Academic Medical Centre, Amsterdam, The Netherlands; 7 Department of Pathology, Academic Medical Centre, Amsterdam, The Netherlands; 8 Department of Cardiology, St. Radboud University Medical Centre, Nijmegen, The Netherlands; 9 Department of Clinical Genetics, St. Radboud University Medical Centre, Nijmegen, The Netherlands; 10 Department of Cardiology, Leiden University Medical Centre, Leiden, The Netherlands; 11 Department of Clinical and Human Genetics, Leiden University Medical Centre, Leiden, The Netherlands; 12 Department of Cardiology, University Medical Centre Groningen, Groningen, The Netherlands; 13 Department of Clinical Genetics, University Medical Centre Groningen, Groningen, The Netherlands; 14 Durrer Cardiogenetic Research Center, Utrecht, The Netherlands; 15 Department of Clinical Genetics and DNA Diagnostics, VU University Medical Centre, Amsterdam, The Netherlands; 16 Department of Clinical Genetics, Academic Medical Centre, Amsterdam, The Netherlands; South Texas Veterans Health Care System and University Health Science Center San Antonio, United States of America

## Abstract

**Background:**

Marfan syndrome (MFS) is a pleiotropic genetic disorder with major features in cardiovascular, ocular and skeletal systems, associated with large clinical variability. Numerous studies reveal an involvement of TGF-β signaling. However, the contribution of tissue inflammation is not addressed so far.

**Methodology/Principal Findings:**

Here we showed that both TGF-β and inflammation are up-regulated in patients with MFS. We analyzed transcriptome-wide gene expression in 55 MFS patients using Affymetrix Human Exon 1.0 ST Array and levels of TGF-β and various cytokines in their plasma. Within our MFS population, increased plasma levels of TGF-β were found especially in MFS patients with aortic root dilatation (124 pg/ml), when compared to MFS patients with normal aorta (10 pg/ml; p = 8×10^−6^, 95% CI: 70–159 pg/ml). Interestingly, our microarray data show that increased expression of inflammatory genes was associated with major clinical features within the MFS patients group; namely severity of the aortic root dilatation (*HLA-DRB1* and *HLA-DRB5* genes; r = 0.56 for both; False Discovery Rate(FDR) = 0%), ocular lens dislocation (RAET1L, CCL19 and HLA-DQB2; Fold Change (FC) = 1.8; 1.4; 1.5, FDR = 0%) and specific skeletal features (*HLA-DRB1*, *HLA-DRB5, GZMK*; FC = 8.8, 7.1, 1.3; FDR = 0%). Patients with progressive aortic disease had higher levels of Macrophage Colony Stimulating Factor (M-CSF) in blood. When comparing MFS aortic root vessel wall with non-MFS aortic root, increased numbers of CD4+ T-cells were found in the media (p = 0.02) and increased number of CD8+ T-cells (p = 0.003) in the adventitia of the MFS patients.

**Conclusion/Significance:**

In conclusion, our results imply a modifying role of inflammation in MFS. Inflammation might be a novel therapeutic target in these patients.

## Introduction

Marfan syndrome (MFS) is an autosomal dominant connective tissue disorder with a systemic involvement. Main clinical features are aortic root dilatation, ocular lens dislocation and typical skeletal features resulting from overgrowth of long bones. Other organ systems like lungs, dura and in tegmentum are also often affected. It has an incidence of approximately 1 in 5,000 and is probably the most common monogenetic connective tissue disorder [1].

MFS is caused by mutations in the fibrillin-1 gene (*FBN1*, MIM 134797) coding for the fibillin-1 protein, which is an abundant component of the extracellular matrix throughout all organ-systems [2], [3]. It has been thought that structural weakness of the connective tissue due to a defect fibrillin-1 protein leads to its clinical manifestations. Recently, evidence emerged about the role of altered Transforming Growth Factor-β (TGF-β) signaling in MFS pathogenesis. The extracellular matrix was found to regulate activation and bioavailability of TGF-β [4]. Due to a defect or deficient fibrillin-1, enhanced release and activation of TGF-β occurs in the extracellular matrix. Both Smad-dependent and non-canonical (ERK dependent) TGF-β signaling contribute to the development of aortic root aneurysm in mouse model of MFS [5], [6]. TGF-β blockade by anti-TGF-β antibodies reduced the aortic root dilatation and myopathy in a mouse model of MFS [7], [8].

Interestingly, losartan, a commonly used anti-hypertensive drug with angiotensin (Ang) II blocking effects, had the same effect as anti-TGF-β antibodies, which offers novel treatment possibilities [7]–[9]. Losartan blocks selectively the angiotensine type 1 (AT1) receptor, allowing the protective angiotensine type 2 (AT2) receptor signaling in Marfan mice [5]. Apart from its role in hypertension, Ang II is a well known pro-inflammatory peptide that effects the cardiovascular system and can even induce aortic aneurysm formation (predominantly in the descending aorta) in mice. TGF-β activity, however, protects against inflammatory aneurysm progression in the murine AngII-induced aneurysm model, which is contradictory to the pathogenic role of TGF-β in MFS [10]. This reflects the complexity of the relation of TGF-β signaling and inflammation during aortic dilatation, which remains elusive in MFS.

Apart from losartan, there are more indications that inflammation plays a role in aortic root dilatation in the Marfan mouse model. Aortic wall extracts of Marfan mice induced increased chemotaxis of macrophages in vitro, mainly induced by elastin binding protein, showing a chemotactic stimulatory activity of fibrillin-1fragments [11]. These results were recently confirmed in MFS patients' aortas.[12] Furthermore, macrophages found in the aortic wall of these mice are positive for certain matrix metalloproteinases, which are enzymes that contribute to the degradation of the extracellular matrix. Doxycycline, a nonspecific inhibitor of matrix metalloproteinases, ameliorated aortic dilatation, improved elastic fiber organization and reduced TGF-β signaling in MFS mice. This suggests that macrophage activation contributes to the microfibril reduction and subsequent aortic dilatation. In addition, in patients with MFS increased counts of inflammatory cells were found in the media of the aortic wall when compared with non-MFS controls, suggesting that an inflammatory process could enhance disease progression [13].

MFS is a pleiotropic disorder with large clinical and genetic variability both between and within MFS families [14], [15]. The variable and unpredictable clinical course of the disease hampers clinical management and counseling of these patients. In this transcriptome wide gene expression study, we investigated the role of TGF-β and inflammation related genes within a group of MFS patients, to elucidate if these pathways are correlated to MFS severity or specific MFS clinical features.

## Results

Microarray analysis was performed with fresh frozen skin biopsies of 55 MFS patients, using Affymetrix Human Exon 1.0 ST Arrays (approximately 17,800 genes). Patients' characteristics are presented in Table 1. Skin was used as a model of the connective tissue, because of the presence of myofibroblasts that produce extracellular matrix proteins in the most voluminous part of connective tissue in our body, namely the skin-dermis.

**Table 1:** Patients characteristics.

Features A	55 MFS Patients
Age,y, mean (range)	36 (18–62)
Sex (male/female)	33/22
**Cardiovascular features**	
Aortic root dilatation^B^	47 (85)
Mean aortic root dilatation rate (SD)	0.46 (0.4)
Mitral valve prolapse	25 (45)
**Ocular lens dislocations**	32 (58)
**Skeletal features**	
Pectus deformity	33 (60)
severe excavatum	10 (18)
mild excavatum	7 (12)
carinatum	16 (30)
Reduced elbow extension	9 (16)
Joints hypermobility	14 (25)
Severe scoliosis	15 (27)
Hindfoot deformity	26 (47)
Wrist and thumb sign	37 (60)
**Other MFS features**	
Dural ectasia	21 (38)
Pneumothorax/apical blebs	11 (20)

A Data are shown in patient numbers (percentage) if not otherwise indicated.

B Of these patients 18 had aortic root replacement and two had dilatation of descending aorta.

Upon transcriptome wide gene expression analysis, patients were clustered based on gene expression profiles using hierarchical clustering. This analysis revealed four distinct patient clusters. (Figure S1). However, disease severity did not differ between the patients clusters (data not shown). This implies that there is no uniform gene expression pattern in the connective tissue of the skin that can predict disease severity or a pattern of clinical MFS features. Gene expression may not be regulated in a patient specific manner, but possibly in an organ specific manner. To test this, we investigated whether different features co-segregated in same patients and found low correlations (r = 0–0.3, data not shown). This supports the hypothesis that the pathogenesis of different phenotypes in MFS is most probably organ specific. In order to identify genes and pathways contributing to the pathogenesis of different clinical features of MFS (concerning specific organ-systems), we compared gene expression profiles per MFS feature within the MFS patient group, where different patients show different MFS features, as listed in Table 1.

### Aortic root dilatation is associated with increased expression of inflammatory genes and enhanced TGF-β levels in blood

**Figure 1:**
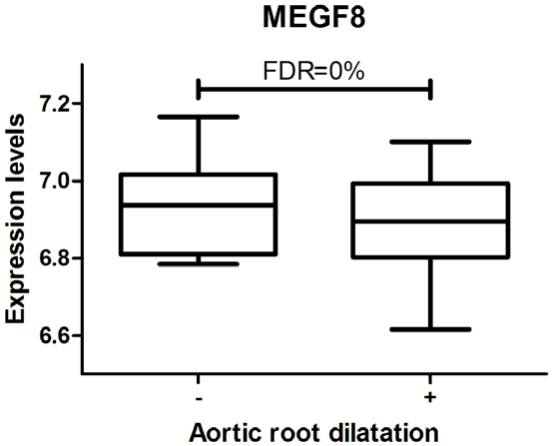
In patients with aortic root dilatation *MEGF8* was found relatively down-regulated (FDR = 0%, FC = 0.62). *MEGF8* contains multiple EGF-like domains which are believed to play an important role in cell-adhesion and receptor-ligand interactions.

Gene expression was first compared between MFS patients with and without aortic root dilatation. It revealed one significantly down-regulated gene in patients with aortic root dilatation: *MEGF8* (Multiple Epidermal Growth Factor-like-domains 8) (FDR = 0%, FC = 0.62) (Figure 1).

**Figure 2:**
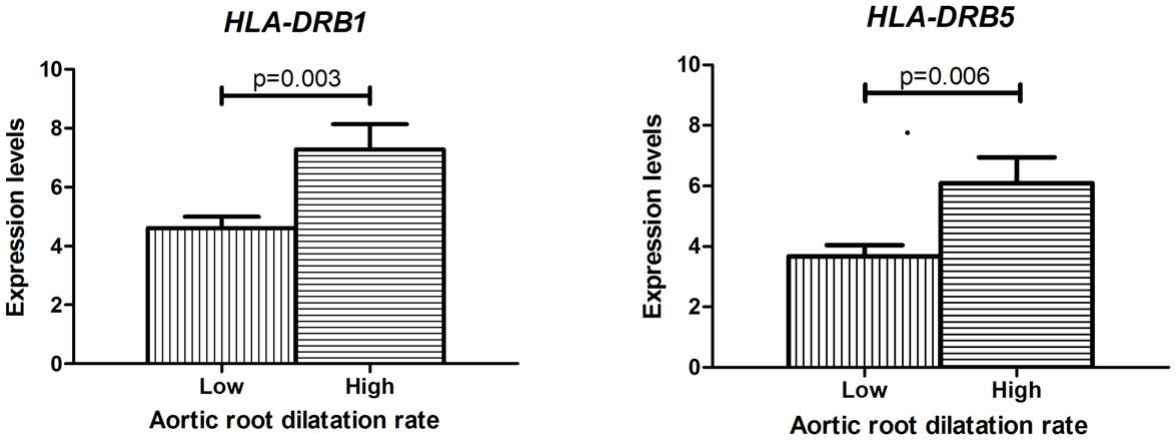
Differences in aortic root dilatation rates measured over a period of 3–12 years between patients with low (0.1 mm/yr) and high (0.9 mm/yr) gene expressions of HLA-DRB1 and HLA-DRB5 genes.

Next, we studied gene expression in the subpopulation of MFS patients who are monitored in time for aortic root dilatation. Aortic phenotype was first analyzed as aortic root dilatation rate (mm/yr) in non-operated patients, which reflects the progressiveness of the disease. Strikingly, patients with progressive aortic root dilatation (mean aortic dilatation rate of 0.9 mm/year) had an increased expression of *HLA-DRB1* and *HLA-DRB5* genes (r = 0.56 for both; FDR = 0%) (Figure 2) when compared to the patients with low aortic root dilatation rate (mean aortic dilatation rate of 0.1 mm/yr). These two genes code for the heavy chain of the MHC II, which mediates activation of T helper cells during an adaptive immune response. Up-regulation of these genes indicates an increased inflammatory response in patients with progressive aortic disease compared to patients with mild aortic disease. Interestingly, whereas the expression of a growth related gene was down-regulated when comparing MFS with versus without aortic dilatation, expression of inflammatory genes was up-regulated in patients with progressive aortic disease.

**Figure 3:**
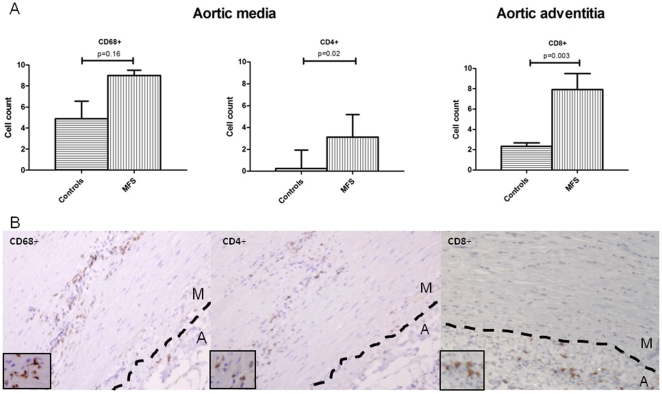
Inflammatory cells present in the aortic root vessel wall. (A) Increased cell counts of macrophages (CD68+) and T helper cells (CD4+) were found in the aortic media of MFS patients when compared to 6 non-Marfan controls. In adventitia, however, we found increased numbers of cytotoxic T cells (CD8+). (B) In the media, the inflammatory cells were segregating around fields of cystic media necrosis (arrows), predominantly along the adventitial side of aortic wall, in MFS patients. In the adventitia, the CD8+ T-cells showed the same segregation pattern along the media-adventitia border. M- aortic media; A- aortic adventitia.

We further compared inflammatory cell-profiles in aortic surgical specimens of 11 MFS patients after aortic root replacement with 6 control aortic root specimens of patients with Marfan-unrelated aortic pathology. Immunohistochemistry was performed using well-established markers of macrophages, T-helper cells and cytotoxic T-cells (CD68+, CD4+ and CD8+, respectively). Aortic media and adventitia were analyzed separately. Histological analysis of Marfan patients' aortic media revealed cystic media necrosis with increased macrophage counts, although not significantly (p = 0.16) (Figure 3A). CD4+T helper cell counts were significantly increased (p = 0.02), whereas the counts of cytotoxic T-cells did not differ between the groups in the aortic media (2 cells/field +/−2; p = 0.4). In the adventitia, however, we found increased counts of cytotoxic T-cells (p = 0.003) (Figure 3A) and no difference in the counts of other cell-types (CD68: 24 cells/field +/−10, p = 0.8; CD4: 18 cells/field +/−13, p = 1). Both in aortic media and adventitia, inflammatory cells segregated along the media/adventitia border (Figure 3B).

**Figure 4:**
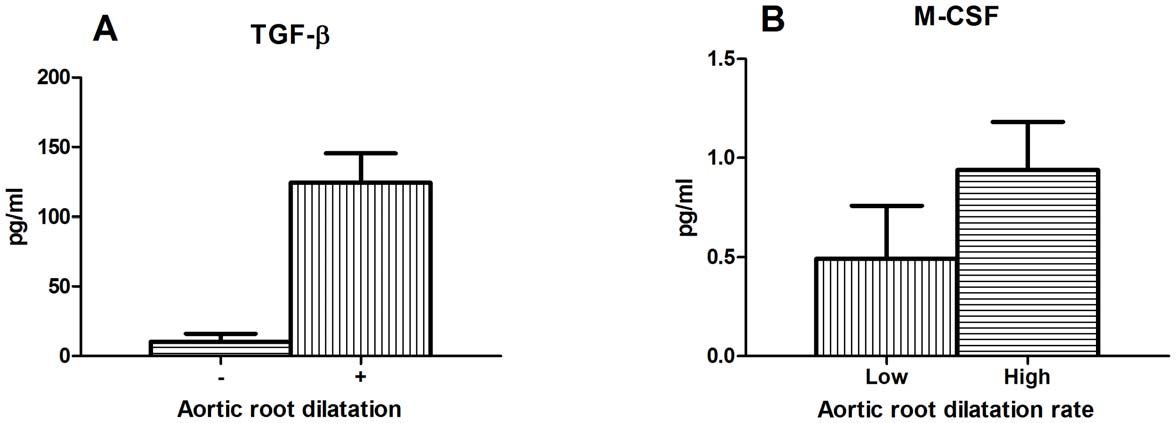
Cytokines in plasma from MFS patients. (**A**) In patients with aortic root dilatation strikingly higher levels of TGF-β were found (p = 8×10^−6^), when compared to the patients without aortic root dilatation. (**B**) The progression of the aortic disease was reflected by the levels of M-CSF. Patients with progressive aortic root dilatation (aortic dilatation rate of 0.9 mm/year) had higher levels of Macrophage-Colony Stimulating Factor (M-CSF) in plasma (p = 0.05) when compared to patients with mild aortic disease (aortic dilatation rate of 0.1 mm/y).

Similarly, we investigated whether TGF-β or inflammatory markers in blood would correlate to aortic phenotype in MFS patients. TGF-β, C-Reactive Protein (CRP; a well established aspecific marker of systemic inflammation) and several prominent cytokines were measured in plasma of our MFS patient group (Table S1). The tested cytokines are pro- or anti-inflammatory in nature and can be produced by activated tissue cells and inflammatory cells during an inflammatory response, causing growth/differentiation and chemoattraction [16]. The average levels of a large number of cytokines was barely detectable (namely IL-1β, IL-2, IL-4, IL-6, IL-10 and TNFα). Certain cytokines had substantially detectable levels in plasma (for example IL-8, IL-17, IL-18, IFN-γ and MCP-1) in a number of patients, however we could not correlate these high levels in these patients to specific MFS features. Interestingly MFS patients with aortic root dilatation had a 10-fold higher level of TGF-β (125 pg/ml) when compared to the patients without aortic root dilatation (p = 8×10^−6^) (Figure 4A). Yet, TGF-β levels did neither correlate with the aortic root diameter when corrected for age, sex and BSA (Z-score of the aortic root), nor with the progressiveness of the aortic dilatation rate (p = 0.2 and p = 1, respectively) (Figure S2A and Figure S2B). In MFS patients, CRP levels were not elevated (Table S1). CRP levels in the normal range (<5 mg/L) are known to be predictive of atherosclerosis-related cardiovascular events such as myocardial infarction or stroke, with moderate risk at levels >1 mg/L and high risk at levels >3 mg/L. CRP levels, however, did not correlate with any features investigated. Of all inflammatory cytokines measured, only Macrophage-Colony Stimulating Factor (M-CSF) correlated with a specific MFS feature, namely progressive aortic root dilatation. Patients with a dilatation rate of 0.9 mm/year had 2-fold higher levels of M-CSF in plasma (1 pg/ml, p = 0.05) when compared with patients with mild aortic disease with an aortic dilatation rate of 0.1 mm/year (Figure 4b). Therefore, the progression of the disease correlated with a potent macrophage attractant, namely M-CSF. Together, these findings point to the role of an increased TGF-β signaling in initiating aortic dilatation, whereas the severity of disease progression seems to be defined by an inflammatory response.

### Other features of MFS are associated with inflammation

We further investigated whether inflammation contributes to the pathogenesis of other Marfan features, such as ocular lens dislocation and skeletal features.

#### Ocular lens dislocation

**Figure 5:**
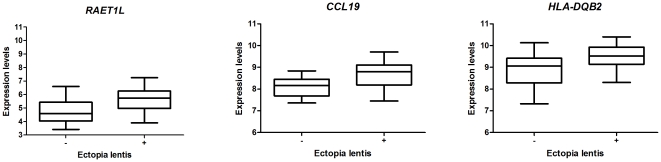
Inflammatory genes significantly up-regulated in patients with ocular lens dislocation. RAETL1 is a member of the MHC I complex, it activates NK and T cells via NKG2D ligand. CCL19 codes for the MIP-3β cytokine which is involved in normal lymphocyte homing and migration. HLA-DQB2 codes for the heavy β chain of the antigen presenting MHC II complex. (FC = 1.8; 1.4; 1.5 respectively, FDR = 0%).

Analysis of differences in gene expression between the patients with ocular lens dislocation and those without ocular lens dislocation revealed eight differentially expressed genes (FDR = 0%) (Table S2). Interestingly, three of these genes were involved in inflammatory pathways, specifically *RAET1L*, *CCL19* and *HLA-DQB2* (Figure 5). These genes were all up-regulated in MFS patients with ocular lens dislocation. *RAETL1* is a member of the MHC I complex family and it activates Natural Killer (NK) cells and T cells. *CCL19* encodes for the MIP-3β cytokine, which is involved in lymphocyte homing and migration. *HLA-DQB2* encodes for the heavy β chain of the antigen presenting MHC II complex. They are members of both the innate and the adaptive inflammatory pathways.

#### Skeletal features

**Figure 6:**
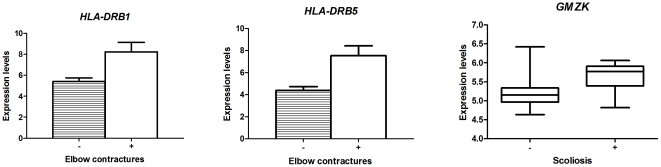
Inflammatory genes up-regulated in patients with reduced elbow extension and scoliosis. HLA-DRB1 and HLA-DRB5 genes code for the heavy chain of the MHC II and are involved in antigen presenting. GZMK gene codes for granzyme K, produced by cytotoxic T-cells and NK cells which induces apoptosis. (FC = 8.8, 7.1 and 1.3 respectively, FDR = 0%).

Furthermore, skeletal features were analyzed. In patients with elbow contractures and scoliosis (sideways curving of the spine), an up-regulation of three inflammatory genes was found when compared with patients without those features, namely *HLA-DRB1* and *HLA-DRB5*, *(*FC = 8.8, 7.1), and *GZMK* (FC = 1.3; FDR = 0%) (Figure 6). The same HLA-DRB genes were also increased in the MFS patients with enhanced aortic dilatation rates. HLA-DRB proteins are involved in MHC-II activation of T-helper cells, while *GMZK* encodes for granzyme K, a cytokine produced by cytotoxic T cells. Cytotoxic T cells were also found to be increased in the adventitia of the MFS aortic vessel wall. The appearance of inflammatory genes in multiple features of MFS strengthens our view that inflammation can play an important role in MFS.

**Figure 7:**
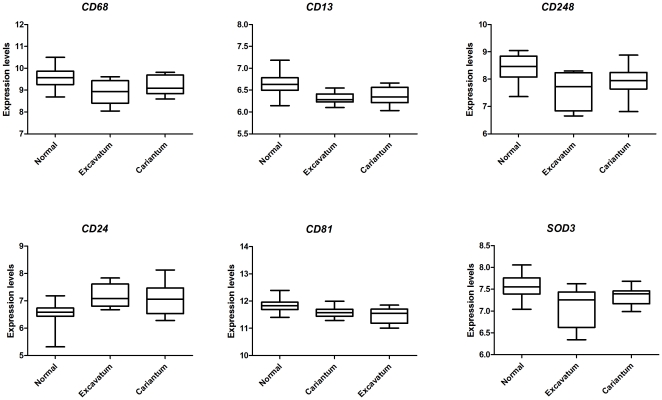
Six inflammatory genes differentially expressed in patients with pectus deformities, of which five are down-regulated. The CD molecules are cell surface molecules participating in inflammatory cell interactions. SOD3 encodes for extracellular superoxide dismutase, which is an enzyme that protects against oxidative stress caused by inflammatory cells. (FC = 0.6, 0.8; 0.6; 1.6; 0.6; 0.6respectively; FDR = 0%).

Interestingly, in patients with chest deformities resulting from the over-growth of ribs, we found a predominant up-regulation of growth-related genes. Expression profiles of patients carrying pectus deformities (severe pectus excavatum and pectus carinatum) were compared with patients without these features. Multiclass analysis of these deformities revealed 40 differentially expressed genes with FDR = 0% between the groups. (Table S3 for up-regulated genes and Table S4 for down-regulated genes). Some of these genes are known TGF-β related genes (FLRT3, WISP2, CLK1, ACVRL1 and IGFBP4). In addition, six inflammatory genes were present in the list of differentially expressed genes, namely CD68, CD13, CD248, CD24, CD81 and SOD3 (Figure 7). The CD molecules are cell surface molecules participating in pro-inflammatory cell interactions. SOD3 encodes for extracellular superoxide dismutase, which is an enzyme that protects against oxidative stress caused by inflammatory cells. (FC = 0.6, 0.8; 0.6; 0.6; 1.6; 0.6 respectively; FDR = 0%). Five of these inflammatory genes were down-regulated in patients with chest deformities when compared to patients without these features. Only CD24 was increased in this subpopulation of MFS patients. CD24 is known to selectively repress tissue damage-induced immune responses [17] and in that light high CD24 is anti-inflammatory.

When looking at a relationship between blood TGF-β levels and chest deformities, no significant correlation was found, even though genes related to TGF-β signaling are differentially regulated strongly in this MFS feature.

MFS patients with other skeletal features, mitral valve prolapse, dural ectasia and pneumothorax revealed no gene expression differences when comparing them to MFS patients without these specific features.

## Discussion

In this study we demonstrate that, next to the established role of TGF-β, there is an association of increased expression of inflammatory genes with aortic root dilatation, ocular lens dislocation and most skeletal features in MFS. Our results introduce a potential aggravating role of inflammation in disease severity which seems to be “on top of” disturbed TGF-β signaling.

All Marfan features develop during the postnatal life, they are age dependent and accompanied by microfibril-reduction on the molecular level. Low-grade inflammation may contribute to this process. It is known that some of these features, like the elbow contractures and spine deformities, can be seen in other inflammatory and auto-immune diseases, such as rheumatoid arthritis and ankylosing spondylitis. Whereas many MFS features showed and increased inflammatory profile, only chest deformities resulting from overgrowth of the ribs showed a reduced inflammatory response and increased TGF-β signaling on a gene expression level.

On protein level, TGF-β plasma values were highly increased in MFS patients with aortic root dilatation. On gene expression level, decreased expression of *MEGF8* gene was found in MFS patients with aortic root dilatation. Although there is little known about the function of this gene, it contains multiple EGF-like domains like *FBN1* gene which are believed to play an important role in cell-adhesion and receptor-ligand interactions. Recently, mutations in this gene were found to result in extensive cardiac malformations in mice and zebrafish (heterotaxy) indicating that this gene is important for normal development of the cardiovascular system [18]. Aortic disease is the main clinical problem in MFS patients and defines the morbidity and the mortality in this patient group. Strikingly, the high levels of TGF-β in blood showed no correlation with the progressiveness of the aortic disease. Inflammation, however, did correlate with progression measured in time as the aortic root dilatation rate. The discrete “on-top-of disturbed TGF-β signaling” contribution of inflammation in the disease pathogenesis might be the reason that MFS was never seen as an inflammatory disease. Now it seems that TGF-β levels in blood could possibly function as a biomarker to predict the onset of an aortic phenotype. In addition, when MFS patients have been diagnosed with aortic root dilatation, increased blood M-CSF levels can indicate the aggressiveness of the aortic disease in order to differentiate MFS patients needing frequent or less frequent follow-up of aortic root diameters. With the knowledge that inflammation plays a key role in the aortic root dilatation rate, an active search can be started to find even stronger biomarkers for disease progression.

On gene expression level, expression levels of *HLA-DRB1* and *HLA-DRB5* genes in the skin could distinguish between patients with mild aortic disease and those with progressive aortic disease. Interestingly, HLA-DRB1 polymorphisms are linked to chronic periaortitis patients (with incidence of aneurysm development) and abdominal aortic aneurysm patients [19]–[22], suggesting these genes have significance in aortic aneurysm formation. SNP analysis of these polymorphisms in large groups of MFS patients may therefore be informative as to which MFS patient is susceptible to develop progressive aortic root dilatation (or elbow contractures).

Our data indicate that inflammatory responses contribute particularly to aortic disease progression and may independently explain the large clinical variability seen in patients carrying even the same *FBN1* gene mutation. Along this line, the aortic root phenotype was clearly improved in Marfan mice when the drugs with additional anti-inflammatory properties (losartan [23], [24] or doxycyclin [25]–[27]) were administered. Even less MFS manifestation was observed when both drugs were given simultaneously, indicating different inflammatory pathways involved in aortic root dilatation (19). The improved MFS phenotype with anti-inflammatory drugs was established in the background of TGF-β signaling in these mice, which should thus also be feasible in human MFS patients in future.

Historically, aortic disease in MFS is considered a disease of the aortic media, where we indeed find medial necrosis and inflammatory cells. However, our histological results also show significantly increased counts of CD8+ T-cells in the Marfan aortas, emphasizing a role for the aortic adventitia. In line with these data, Lindeman and co-workers found that collagen microarchitecture in the aortic adventitia of MFS patients was impaired, whereas the medial layer is relatively intact when it comes to endurance of pressure [28]. Basically, the adventitia serves as an “external stent” determining maximal diameter of the vessel. When its structure is impaired its “stent” function is lost. The authors also showed that fibrillin-1 is more abundantly present in the adventitia of healthy control tissue (where it is associated to collagen fibers), rather than in the media of the aortic wall (20). Therefore, it seems that *FBN1* mutations could easily affect the organization of the collagen fibers in the aortic adventitia. TGF-β signaling is known to enhance collagen production, however, the quality of the collagen triple helix and the network of fibers formed is presumably more important that the amount of collagen alone. Defective fibrillin-1 in MFS apparently leads to an impaired collagen fiber/network organization. Aortic damage due to impaired resistance to pressure may cause collagen damage, and collagen degradation products resulting from this process may induce inflammation. The susceptibility of certain MFS patients to develop aortic inflammation remains to be elucidated.

The main limitation of this study is its correlational character, as biological material obtained allowed no functional studies. Our work provided interesting directions for future comprehensive research on interactions of TGF-β and inflammatory pathways.

In conclusion, the most prominent finding of this paper is a simultaneous increase of TGF-β and inflammation in aortic dilatation in Marfan syndrome, with TGF-β being predictive for aortic dilatation and inflammation being correlated to the progression of aortic root dilatation. In addition, we presented evidence of inflammatory contribution in the development of many of the other clinical features of MFS, with the exception of chest deformities. These findings open novel possibilities for anti-inflammatory treatment strategies in Marfan patients to reduce MFS severity.

## Methods

### Study subjects, phenotype and biological material

Patients were adult patients with an established diagnosis of MFS according to the Ghent criteria, participating in the COMPARE study [29]. All the analyses presented in this study were performed in the biological material collected at the study inclusion, prior to losartan initiation. The COMPARE study is a randomized clinical trial investigating effects of losartan on a wide range of clinical and molecular parameters in MFS patients. Phenotypic features were scored at the inclusion in the COMPARE study by two investigators as indicated for the diagnosis of the MFS according to the Ghent criteria [30]. Aortic root dilatation was assessed by means of cardiac Magentic Resonance (cMRI) as described earlier [31]. Aortic root dilatation rate (mm/y) was derived first in a discovery set of 20 patients in whom aortic root dilatation rate was available from a period of 12 years of follow-up with cMRI. In additional 13 patients aortic root dilatation rate was derived from clinical cMRI scans over a period of at least 3 years. Punch skin biopsies were taken from the upper thigh of consenting patients and immediately snap-frozen. Biological material sampling and the trial were conducted with approval from institutional review boards in four participating academic hospitals in the The Netherlands (Academic Medical Centre Amsterdam; Radboud University Nijmegen Medical Centre; Leiden University Medical Centre; University Medical Centre Groningen). Written informed consent was obtained from all participants.

### RNA isolation

Full skin punch biopsies of 4 mm (∼5–15 mg) were used to isolate total RNA. Skin biopsies were pulverized in liquid nitrogen and transferred to 1.5 ml tubes containing Qiazol (Qiagen). Crude RNA extractions were obtained according to manufacturer's instructions with the addition of Phase-Lock Gel Heavy (5 Prime) to obtain a better phase separation. The crude RNA fractions were further purified with the RNeasy Minelute Cleanup Kit (Qiagen) according to Appendix D protocol: RNA Cleanup after Lysis and Homogenization with QIAzol Lysis Reagent. RNA yield was measured on a Nanodrop ND-1000 (Thermo Fisher Scientific) and the RNA quality was investigated on the BioAnalyzer 2100 (Agilent Technologies) with the RNA 6000 Pico Chip Kit (Agilent Technologies). Only RNA samples with sufficient yield and RIN-values above 6.5 were used for analysis.

### Microarrays

Gene expression was analyzed with Affymetric Human Exon 1.0 ST Arrays in 55 patients. Sense-strand cDNA was generated from total RNA using Ambion WT Expression Kit (Applied Biosystems) conform manufacturer's instructions. Further steps were performed using manufacturer's protocols for the GeneChip platform (Affymetrix). Those included purification of double-stranded cDNA, synthesis of cRNA by in vitro transcription, recovery and quantitation of biotin-labeled cRNA, fragmentation of this cRNA and subsequent hybridization to the microarray slide, posthybridization washings and detection of the hybridized cRNA using a streptavidin-coupled fluorescent dye. Hybridized Affymetrix Arrays were scanned using Gene-Chip Scanner 3000-7G (Affymetrix). Image generation and feature extraction were performed using Affymetrix GCOS Software v1.4.0.036.

### TGF-β and cytokine measurements

Cytokines and TGF-β were assessed in EDTA plasma samples. Total TGF-β1, i.e. latent and active TGF-β, was determined with an ELISA using commercially available kit (Bio-Rad, Richmond, CA). The neo-epitope in active TGF-β that is recognized by the antibody pair is induced in latent TGF-β1 by acid-activation (with 1N HCl). Inflammatory mediators were measured using the custom suspension bead assays (Bio-Rad, Richmond, CA) using a Luminex reader.

### Immunohistochemistry of aortic specimen

Aortic tissue of MFS patients was collected from operated patients in the period of 2008–2010 and was immediately formalin-fixated. Aortic tissue of six control patients was gathered from the pathology records of AMC Amsterdam. Primary aortic pathology in six control aortic samples (age range 21–68 years) was unrelated to MFS: four of controls were surgical specimen and two were autopsy material. Autopsy specimens were from two patients who died from subarachnoidal bleeding and myocardial infarction. Surgical specimens were: post-stenotic aortic root dilatation; aspecific atherosclerosis of aorta; degenerative aortic dilatation; bicuspid aortic valve malformation without cystic media necrosis. Patients and control aortic samples were paraffin-embedded and immunohistochemical staining was performed using widely used and established antibodies, detecting CD+4, CD8+ and CD68+ markers. Cells were counted in the aortic media and adventitia.

### Statistical analysis and functional annotation

Hierarchical clustering of microarray data was performed in R program using “stats” package. Differences in clinical features between patients in different clusters were investigated using ANOVA and Chi-square tests in R program using “stats” package. T-test was used to investigate cytokine differences between groups of patients in “stats” package. Differences in gene expression between patients were analyzed using univariate linear regression analysis with permutation testing in Significance Analysis of Microarrays R package in R program [32]. Only genes with a q-value (FDR) of 0 were considered significant. Gene functions presented in tables were searched manually in NCBI databases; only direct experimental evidence or associations from genome wide association studies were included in functional annotation reported in tables.

## Supporting Information

Figure S1 Heatmap of the hierarchical clustering of 55 MFS patients based on similar gene expression patterns. Four distinct patients' clusters of 16, 27, one and 11 patients were defined. Approximately 1800 genes differed significantly (FDR = 0%, minimal fold change = 2) between the clusters. However, clinical significance of these findings seems to be limited as no differences were found in disease severity between the patients' clusters.
(DOC)

Figure S2 Plots of the correlation of (A) Z-score (aortic root diameter corrected for age, sex and Body Surface Area) and (B) aortic root dilatation rate with TGF-β levels in plasma of MFS patients. None of the two parameters of progressiveness of aortic disease correlated with TGF-β (p = 0.2 and 1 respectively).
(DOC)

Table S1 Plasma levels of TGF-β, CRP, and cytokines in MFS patients. Note: A – mean levels of cytokines in plasma of patients. B – mean level of CRP (mg/L) in patients serum.
(DOC)

Table S2 Genes differentially expressed in patients with and without ectopia lentis. Note: A- Fold change, a ratio between mean expression levels in patients with ectopia lentis and ones without it.
(DOC)

Table S3 Up-regulated genes in patients with pectus deformities. Note: A- FC exc –ratio between mean expression levels of patients with severe pectus excavatum and patients without pectus deformity. B- FC car- ratio between mean expression levels of patients with pectus carinatum and patients without pectus deformity.
(DOC)

Table S4 Down-regulated genes in patients with pectus deformities. Note: A-FC exc –ratio between mean expression levels of patients with severe pectus excavatum and patients without pectus deformity. B- FC car- ratio between mean expression levels of patients with pectus carinatum and patients without pectus deformity.
(DOC)
