# Supplementary figures and images for: Inflammation Aggravates Disease Severity in Marfan Syndrome Patients
Source: PLoS One. 2012 Mar 30;7(3):e32963. doi: 10.1371/journal.pone.0032963 (PMC3316543; doi:10.1371/journal.pone.0032963)

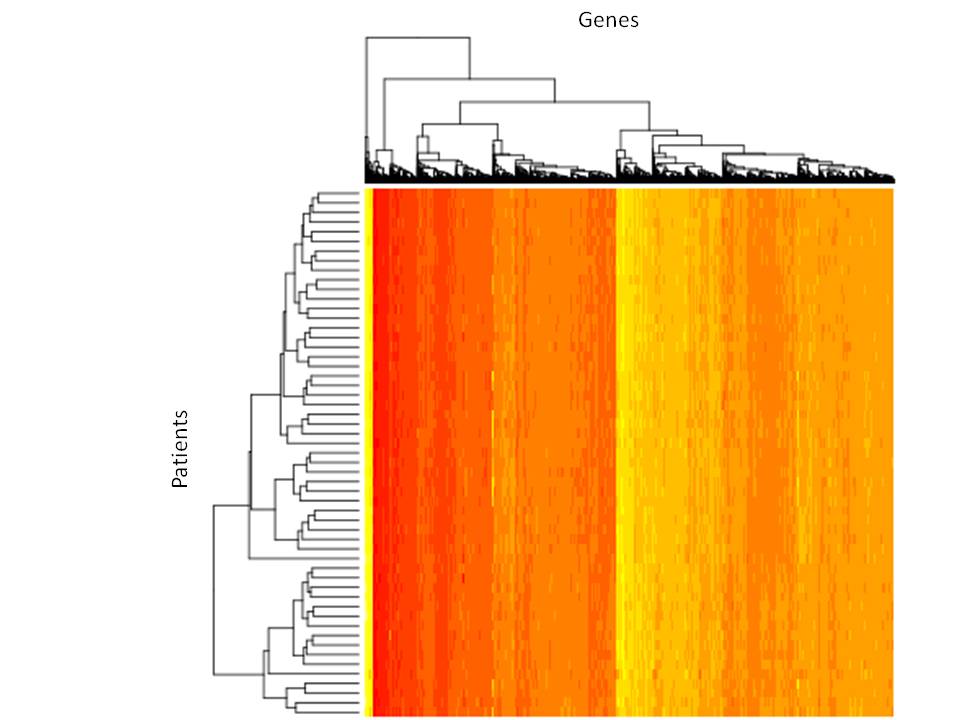

Supplement: Figure S1 — Heatmap of the hierarchical clustering of 55 MFS patients based on similar gene expression patterns. Four distinct patients' clusters of 16, 27, one and 11 patients were defined. Approximately 1800 genes differed significantly (FDR = 0%, minimal fold change = 2) between the clusters. However, clinical significance of these findings seems to be limited as no differences were found in disease severity between the patients' clusters. (DOC) [file pone.0032963.s001.doc]

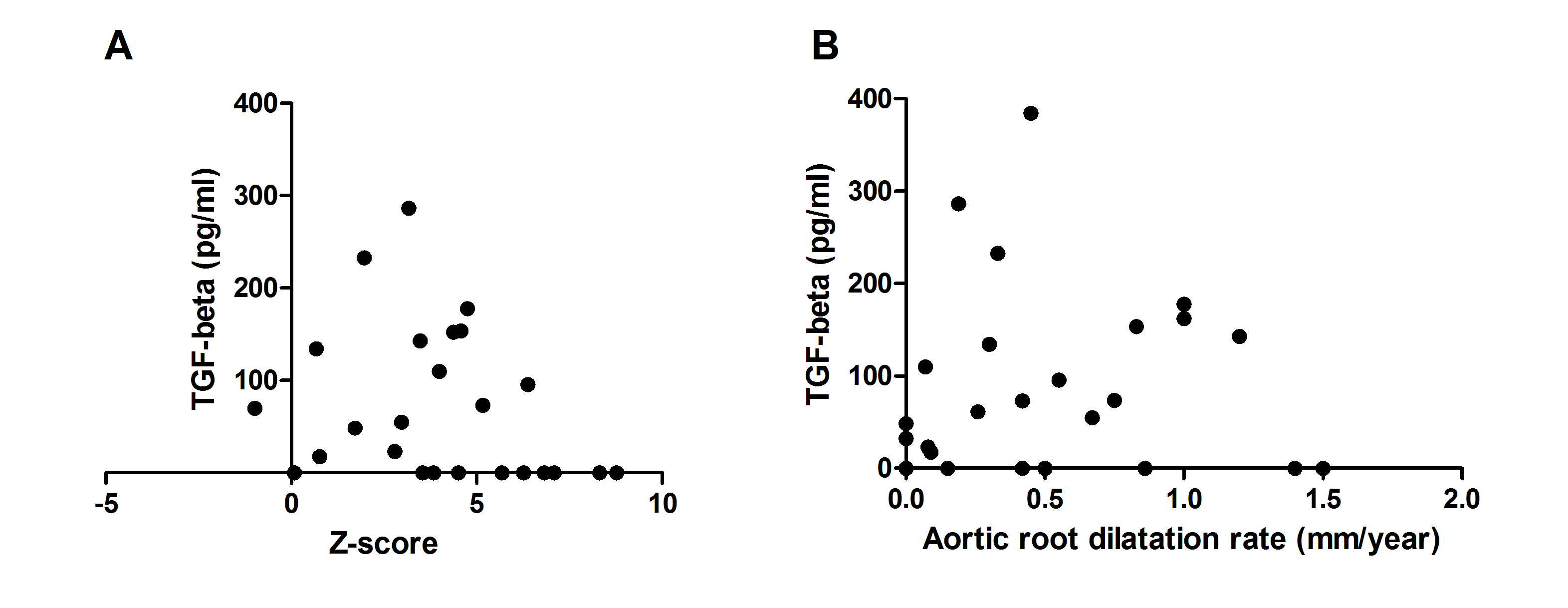

Supplement: Figure S2 — Plots of the correlation of (A) Z-score (aortic root diameter corrected for age, sex and Body Surface Area) and (B) aortic root dilatation rate with TGF-β levels in plasma of MFS patients. None of the two parameters of progressiveness of aortic disease correlated with TGF-β (p = 0.2 and 1 respectively). (DOC) [file pone.0032963.s002.doc]
