# Supplementary material for: Inflammation Aggravates Disease Severity in Marfan Syndrome Patients
Source: PLoS One. 2012 Mar 30;7(3):e32963. doi: 10.1371/journal.pone.0032963 (PMC3316543; doi:10.1371/journal.pone.0032963)
Supplement: Table S1 — Plasma levels of TGF-β, CRP, and cytokines in MFS patients. Note: A – mean levels of cytokines in plasma of patients. B – mean level of CRP (mg/L) in patients serum. (DOC) [file pone.0032963.s003.doc]

Table S1 Plasma levels of TGF-β, CRP, and cytokines in MFS patients.

| **Cytokine** | **pg/ml^A^** | **SD** | **Cytokine** | **pg/ml^A^** | **SD** | **Cytokine** | **pg/ml^A^** | **SD** |
| --- | --- | --- | --- | --- | --- | --- | --- | --- |
| **TGF-β** | 112.08 | 119.6 | **IL6** | 0.36 | 0.96 | **IL18** | 45.57 | 24.7 |
| **CRP** | 2.26**^B^** | 4.98 | **IL8** | 6.52 | 37.3 | **IFN-ϒ** | 6.03 | 16.2 |
| **IL1-β** | 0.04 | 0.15 | **IL10** | 0.05 | 0.2 | **MCP-1** | 14.37 | 14.3 |
| **IL2** | 0.43 | 1.34 | **IL12** | 1.20 | 3.6 | **TNF-α** | 0 | 0 |
| **IL4** | 0.01 | 0.06 | **IL17** | 32.04 | 191.3 | **M-CSF** | 0.92 | 1.3 |

A – mean levels of cytokines in plasma of patients

B – mean level of CRP (mg/L) in patients serum
