# Supplementary material for: Inflammation Aggravates Disease Severity in Marfan Syndrome Patients
Source: PLoS One. 2012 Mar 30;7(3):e32963. doi: 10.1371/journal.pone.0032963 (PMC3316543; doi:10.1371/journal.pone.0032963)
Supplement: Table S2 — Genes differentially expressed in patients with and without ectopia lentis. Note: A- Fold change, a ratio between mean expression levels in patients with ectopia lentis and ones without it. (DOC) [file pone.0032963.s004.doc]

Table S2 Genes differentially expressed in patients with and without ectopia lentis

| **Gene** | **FC^A^** | **Function** | **FDR (%)** |
| --- | --- | --- | --- |
| UNC93A | 1.5 | - | 0 |
| FAM83D | 1.3 | Mitotic spindle protein | 0 |
| RAET1L | 1.8 | Member of the MHCI complex; activates NK and T cells via NKG2D ligand | 0 |
| CCL19 | 1.4 | Normal lymphocyte homing and migration | 0 |
| C5orf46 | 1.3 | - | 0 |
| HLA-DQB2 | 1.5 | Antigen presenting; heavy chain of MHCII complex | 0 |
| SDR9C7 | 1.3 | - | 0 |
| HIST1H3B | 1.4 | Activation of immediate-early gene transcription | 0 |

A: Fold change, a ratio between mean expression levels in patients with ectopia lentis and ones without it
