# Supplementary material for: Inflammation Aggravates Disease Severity in Marfan Syndrome Patients
Source: PLoS One. 2012 Mar 30;7(3):e32963. doi: 10.1371/journal.pone.0032963 (PMC3316543; doi:10.1371/journal.pone.0032963)
Supplement: Table S3 — Up-regulated genes in patients with pectus deformities. Note: A- FC exc –ratio between mean expression levels of patients with severe pectus excavatum and patients without pectus deformity. B- FC car- ratio between mean expression levels of patients with pectus carinatum and patients without pectus deformity. (DOC) [file pone.0032963.s005.doc]

Table S3 Up-regulated genes in patients with pectus deformities

| **Gene** | **FC exc^A^** | **FC car^B^** | **Function** | **FDR (%)** |
| --- | --- | --- | --- | --- |
| FLRT3 | 2.0 | 1.5 | TGF-β signaling-mediated morphogenesis | 0 |
| SFRS7 | 1.5 | 1.3 | Pre-mRNA splicing factor; part of the spliceosome | 0 |
| DSG3 | 1.7 | 1.4 | Mediates cell-cell junctions; part of desmosomes and cadherin cell adhesion family | 0 |
| ZNF138 | 1.5 | 1.5 | - | 0 |
| MPHOSPH6 | 1.5 | 1.1 | Exosome-associated RNA-binding protein involved in rRNA maturation | 0 |
| CLK1 | 1.6 | 1.5 | Indirect role in TGF-β1 induced splice site selection during pre-mRNA processing | 0 |
| ACAT2 | 1.7 | 1.7 | Lipid metabolism | 0 |
| PEX3 | 1.6 | 1.4 | Peroxisome biosynthesis and integrity | 0 |
| FAM111B | 1.4 | 1.2 | - | 0 |
| PIK3C2G | 1.6 | 1.5 | Phosphoinositide 3-kinase (PI3K) family; involved in cell proliferation and survival, oncogenic transformation | 0 |
| C6orf105 | 2.2 | 2.1 | Associated with non-syndromic cleft palate | 0 |
| GEN1 | 1.4 | 1.3 | - | 0 |
| ACADM | 1.8 | 2.1 | Mitochondrial fatty acid beta-oxidation pathway | 0 |
| CD24 | 1.6 | 1.5 | Inhibition of nuclear factor kappaB during innate immune responses | 0 |
| TMEM168 | 1.6 | 1.3 | - | 0 |
| ATL2 | 1.4 | 1.3 | - | 0 |
| GPR87 | 1.7 | 1.4 | Cell-cell communiation; overexpressed in squamous carcinomas | 0 |
| NOP58 | 1.5 | 1.3 | Modification of rRNA via snoRNAs | 0 |
| TMPRSS11E | 2.8 | 3.0 | - | 0 |
| CA13 | 1.5 | 1.3 | Family of zinc metalloenzymes | 0 |

A: FC exc –ratio between mean expression levels of patients with severe pectus excavatum and patients without pectus deformity

B: FC car- ratio between mean expression levels of patients with pectus carinatum and patients without pectus deformity
