# Supplementary material for: Inflammation Aggravates Disease Severity in Marfan Syndrome Patients
Source: PLoS One. 2012 Mar 30;7(3):e32963. doi: 10.1371/journal.pone.0032963 (PMC3316543; doi:10.1371/journal.pone.0032963)
Supplement: Table S4 — Down-regulated genes in patients with pectus deformities. Note: A-FC exc –ratio between mean expression levels of patients with severe pectus excavatum and patients without pectus deformity. B- FC car- ratio between mean expression levels of patients with pectus carinatum and patients without pectus deformity. (DOC) [file pone.0032963.s006.doc]

Table S4 Down-regulated genes in patients with pectus deformities

| **Gene** | **FC exc^A^** | **FC car^B^** | **Function** | **FDR (%)** |
| --- | --- | --- | --- | --- |
| IGFBP4 | 0.7 | 0.8 | Antagonism of insulin-like growth factors I and II | 0 |
| WISP2 | 0.6 | 0.7 | Member of the connective tissue growth factor family; anti-TGFβ signaling effects in myocardium | 0 |
| HSPB6 | 0.6 | 0.7 | Associates with actin and mediates smooth muscle cell relaxation | 0 |
| IFI35 | 0.8 | 0.8 | Mediates cell differentiation, apoptosis and cytoskeleton regulation in interaction with CKIP1 | 0 |
| FXYD5 | 0.8 | 0.9 | Reduction of cell adhesions via down-regulation of E-cadherin | 0 |
| CD68 | 0.6 | 0.7 | Macrophages marker; Member of scavenger receptor family | 0 |
| SLC17A7 | 0.8 | 0.8 | Neurotransmission via glutamate vesicle transport | 0 |
| MFAP4 | 0.5 | 0.8 | Microbifril-associated protein involved in cell adhesions and interactions | 0 |
| XPNPEP2 | 0.6 | 0.8 | Regulates levels of aminopeptidase P in bradykinin metabolism and angioedema | 0 |
| PTGIS | 0.5 | 0.7 | Catalyzes the conversion of prostglandin H2 to prostacyclin (prostaglandin I2) | 0 |
| ACVRL1 | 0.7 | 0.8 | Type I cell-surface receptor for the TGF-beta superfamily | 0 |
| CD81 | 0.8 | 0.8 | Member of the transmembrane 4 superfamily; mediates signal transduction events | 0 |
| CD13 | 0.6 | 0.9 | Cell surface molecules; induce and participate in critical inflammatory cell interactions | 0 |
| RAMP2 | 0.7 | 0.8 | Induces vasorelaxation by adrenomodulin in myocytes | 0 |
| TCN2 | 0.7 | 0.8 | B12-binding protein family; associated with obesity and homocysteine levels | 0 |
| CD248 | 0.6 | 0.7 | Fibroblast cell marker; expressed in proliferating tissues; contributes to fibroblasts proliferation and inflammation | 0 |
| GABRB2 | 0.7 | 0.8 | Encodes neurotrasmitter GABA A receptor, beta 2 subunit | 0 |
| PRELP | 0.6 | 0.8 | Binds type I collagen to basement membranes and type II collagen to cartilage in ECM | 0 |
| SOD3 | 0.7 | 0.8 | Anti-oxidative stress protein; inflammatory cytokine and adhesion molecule expression inhibition | 0 |
| TCIRG1 | 0.8 | 0.8 | Bone morphogenesis; mutations in this gene cause osteopetrosis, excessive bone mineralization | 0 |

A: FC exc –ratio between mean expression levels of patients with severe pectus excavatum and patients without pectus deformity

B: FC car- ratio between mean expression levels of patients with pectus carinatum and patients without pectus deformity
